# Polymer Conformation under Confinement

**DOI:** 10.3390/polym9020073

**Published:** 2017-02-20

**Authors:** Stavros Bollas, Kiriaki Chrissopoulou, Konstantinos S. Andrikopoulos, George A. Voyiatzis, Spiros H. Anastasiadis

**Affiliations:** 1 Institute of Electronic Structure and Laser, Foundation for Research and Technology, Hellas, P.O. Box 1527, 711 10 Heraklion Crete, Greece; stbollas@gmail.com; 2 Institute of Chemical Engineering Sciences, Foundation for Research and Technology, Hellas, P.O. Box 1414, 265 04 Patras, Greece; candrik@iceht.forth.gr (K.S.A.); gvog@iceht.forth.gr (G.A.V.); 3 Department of Chemistry, University of Crete, P.O. Box 2208, 710 03 Heraklion Crete, Greece

**Keywords:** polymer nanocomposites, layered silicates, intercalation, confinement, chain conformation

## Abstract

The conformation of polymer chains under confinement is investigated in intercalated polymer/layered silicate nanocomposites. Hydrophilic poly(ethylene oxide)/sodium montmorillonite, PEO/Na^+^-MMT, hybrids were prepared utilizing melt intercalation with compositions where the polymer chains are mostly within the ~1 nm galleries of the inorganic material. The polymer chains are completely amorphous in all compositions even at temperatures where the bulk polymer is highly crystalline. Attenuated total reflectance-Fourier transform infrared spectroscopy (ATR-FTIR) is utilized to investigate the conformation of the polymer chains over a broad range of temperatures from below to much higher than the bulk polymer melting temperature. A systematic increase of the *gauche* conformation relatively to the *trans* is found with decreasing polymer content both for the C–C and the C–O bonds that exist along the PEO backbone indicating that the severe confinement and the proximity to the inorganic surfaces results in a more disordered state of the polymer.

## 1. Introduction

The growing use of polymers results in a continuous demand of improving their properties as well as of understanding the mechanisms, which rule their response. One way to achieve these aspects is the development of polymer nanohybrids, which, by combining organic and inorganic components, can improve the properties of the initial materials and can provide alternative ways for the understanding of the behavior in a variety of scientific fields [1,2,3,4,5,6,7,8,9,10,11,12,13,14]. The properties of such nanohybrids depend not only on the properties of their individual components but mainly on their morphology and interfacial characteristics [15,16,17]. It is common understanding that even small amounts of nanofiller loadings can significantly affect the properties of the polymer matrix.

The nanofillers utilized in polymer nanocomposites can vary in their dimensionality, i.e., in how many of their dimensions are in the nanometer range, e.g., nanoparticles, nanotubes, or nanorods and layered materials. Of particular interest among the different organic/inorganic nanohybrids are polymer/layered silicate nanocomposites, which constitute a class of materials that has attracted growing scientific and technological interest over the last decades due to their unique properties, which make them candidates for a number of potential applications [18,19,20,21,22,23,24,25,26,27,28,29]. Depending on the polymer/inorganic interactions, the polymer/layered silicate nanohybrids exhibit distinctly different morphologies. In one case, polymer chains penetrate within the interlayer galleries of the inorganic material forming a highly ordered arrangement of alternating organic and inorganic layers; this is the intercalated structure leading to the formation of very thin polymer films of 0.8–2.5 nm thickness. When the interactions between the polymer and the surfaces are very favorable, the registry of the clay particles is destroyed resulting in dispersed platelets within the polymeric matrix; this constitutes the exfoliated structure. In the case of lack of favorable interactions, the two components are phase separated and a microcomposite is formed. Improvement of the properties of the hybrids is usually observed for intercalated [30,31,32,33,34] or exfoliated systems [35,36,37,38,39,40]. Moreover, intercalated nanohybrids are model systems for the investigation of the static and dynamic properties of macromolecules in nano-confinement, which can, however, be investigated utilizing macroscopic samples and conventional analytical techniques [30,31,32,41,42,43,44,45,46,47,48,49,50,51]. The efficient and homogeneous dispersion of the nanofillers within a polymer matrix is closely related to the nature of the initial components. In the case of polar hydrophilic layered silicates, the interactions are favorable with polar polymers like, e.g., poly(ethylene oxide) [31,48,52,53,54]. For hydrophobic polymers, nanocomposites with intercalated or exfoliated structure can be developed only with organophilized clays, i.e., with materials where the hydrated cations within the galleries are replaced by proper surfactants via a cation exchange reaction [44,46,51,55,56,57]. Moreover, the addition of compatibilizers is necessary to enhance the dispersability in cases when less polar polymers, like polyolefins, are used [20,35,37,38,58].

The optimization of properties in the nanohybrids, however, does not depend only on the structure and the state of dispersion of the inorganic additive. There are many cases where controversial results have been reported concerning mechanical or thermal properties despite the fact that an exfoliated structure had been achieved. It is, therefore, necessary to investigate the effect of other factors like, for example, the preparation and processing methods [59,60], as well as the morphology and structure [31] of the polymer itself, which might eventually be altered, in order to fully understand the complex structure-properties relation.

In an earlier work of ours [31], the structure of poly(ethylene oxide) (PEO), chains in polymer/layered silicate nanocomposites was investigated utilizing different experimental techniques. In all cases, intercalated nanohybrids were obtained, whereas an abrupt transition from almost 70% to zero crystallinity was observed at ~70 wt % PEO. That indicated that it was only the excess polymer outside the galleries of the inorganic material that was able to crystallize and that the intercalated polymer chains as well as chains that were in close proximity to the outer surfaces of the inorganic particles remained purely amorphous. It should be mentioned that crystallinity in such nanohybrid materials derives from two competing effects: the enhancement of crystallization in the presence of inorganic fillers [61] and its hindrance due to the severe confinement of the polymer chains and the strong coordination of ether oxygens with the alkali cations present in the interlayer gallery [31,62,63].

PEO is a non-ionic, polar, semicrystalline polymer with high degree of crystallinity. It has attracted scientific interest because it combines specific properties, like water solubility, biocombatibility and biodegradability. At the same time, its simple structure and easy processing makes it a possible candidate for many applications, like, for example, in pharmaceuticals, in medicine as a polar electrolyte and in bioengineering [64,65,66] as well as for the development of solid polymer electrolytes with high ionic conductivity [67,68,69]. Despite the increased interest in PEO and its nanocomposites, there are still open issues concerning its structure, i.e., crystallinity and chain conformation in the proximity of inorganic surfaces. In PEO/layered silicate nanocomposites, the preservation of the PEO helical structure inside the galleries with the cations located in the center of the helix was proposed [70], whereas in another case a highly distorted helical structure was reported to be a better description [59]. A distorted helical conformation and a single-layer arrangement were suggested as a more accurate way to describe the conformation of the PEO chains within the clay galleries as well [71]. Alternatively, it was suggested that the PEO chains within the galleries are not helical but they resemble single or double adsorbed polymer layers onto the clay surfaces [72]. In a more recent work, the *gauche* conformations of the C–C and the C–O bond in a PEO/layered silicate nanohybrid with 30 wt % polymer were significantly increased compared to those in the bulk polymer [31]. More disordered PEO chains were reported in intercalated PEO/montmorillonite systems, as well [73]. PEO confined either in nanopores [74] or between the layers of graphite oxide [75] was found to exhibit a preferential planar zig-zag conformation. Moreover, computer simulations have suggested that intercalated PEO chains are in a liquid-like state and that they are less ordered than the more disordered bulk system due to the strong confinement and the coordination of the ether oxygen with the alkali cations in the galleries [63,76].

In the present work, we investigate in detail the chain conformation in hydrophilic poly(ethylene oxide)/sodium-montmorilonite nanocomposites. The hybrids were prepared utilizing melt intercalation at compositions where the polymer chains are mostly within the ~1 nm galleries of the clay particles. The intercalated structure of the hybrids was verified with X-ray diffraction, which showed that the chains exist in amorphous mono- and bi-layers within the galleries. Attenuated total reflectance-Fourier transform infrared spectroscopy (ATR-FTIR) was utilized to probe the chain conformation as a function of the nanohybrid composition and showed that there is an increase of the *gauche* conformation of both the C–C and the C–O bonds. This increase is systematic with composition for the C–C bond, whereas for the C–O bond there is a clear increase of the most favorable conformation from *trans* to *gauche* but the percentage of the *gauche* C–O population does not change with composition. 

## 2. Materials and Methods

### 2.1. Materials

Poly(ethylene oxide), PEO, purchased from Sigma Aldrich (St. Louis, MO, USA), was used in this study with an average molecular weight of 100,000 g/mol and a polydispersity index *M*_w_/*M*_n_ = 2.4 as determined by Size Exclusion Chromatography with polystyrene standards. The PEO glass transition temperature is *T*_g_ = −67 °C and its melting temperature is *T*_m_ = 65 °C. The inorganic material was a sodium montmorillonite, Na^+^-MMT, with the commercial name Cloisite Na^+^ (Southern Clay, Gonzales, TX, USA). It is a common hydrophilic layered silicate with a cation exchange capacity, CEC, of 92.6 mmol/100 g. The lateral dimensions of the montmorillonite tactoids are in the range of 0.5–2 μm. The existence of sodium cations within the galleries counterbalances the negative charges within the platelet walls due to isomorphic substitution. Na^+^-MMT was used following heating at 120 °C overnight in a vacuum oven to allow removal of excess water molecules from the hydrophilic galleries. PEO/Na^+^-MMT nanocomposites were synthesized utilizing melt intercalation; the two components were mixed in the appropriate amounts, ground in a mortar to get a fine powder and annealed in a vacuum oven at 100 °C for two days. Following the melt intercalation, the temperature for all specimens decreased from 100 °C to room temperature very slowly to ensure equilibrium and the highest degree of crystallinity.

### 2.2. Experimental Techniques

**X-ray Diffraction (XRD):** X-ray diffraction (XRD) was performed for the structural characterization of the pure materials and of the nanohybrids using a RINT-2000 Rigaku diffractometer (Rigaku, Tokyo, Japan). The X-rays are produced by a 12 kW rotating anode generator with a Cu anode equipped with a secondary pyrolytic graphite monochromator. The wavelength of the CuKα radiation used is λ = 1.54 Å. Measurements were performed for diffraction angles 2θ from 1.5° to 30° with step of 0.02°. Materials with periodic structure like the layered silicate clays show characteristic *(00l)* diffraction peaks, which are related to the spacing of the layers according to Bragg’s law, *n*λ *= 2d_00l_*sinθ, where λ is the wavelength of the radiation, *d_00l_* is the interlayer distance, *2*θ is the diffraction angle and *n* the order of the reflection. In the case of an intercalated system, in which the polymer has entered the inorganic galleries causing an increase of the interlayer distance, this main peak is found shifted towards lower angles. If the structure is exfoliated, however, the ordered structure of the silicate is destroyed and, thus, no peaks are observed in the XRD diffractograms.

**Attenuated Total Reflectance-Fourier Transform Infrared Spectroscopy (ATR-FTIR)**: ATR-FTIR was utilized to investigate the chain conformations of the polymer in bulk and in the nanohybrids. FTIR measurements were carried out with an Equinox 55 Bruker spectrometer (Bruker, Billerica, MA, USA) equipped with a DTGS detector (the resolution was set at 3 cm^−1^). A single reflection diamond Golden Gate ATR accessory (Specac, Orpington, UK) was utilized for the collection of high temperature spectra under inert atmosphere. The temperature was controlled with accuracy better than ±1° whereas the inert atmosphere was achieved by flushing the sample with nitrogen for 30 min before every measurement. For correction purposes, a background spectrum was collected for every temperature. Special care was given so that no bands associated with CO_2_ or H_2_O were present after the background correction.

## 3. Results and Discussion

**Figure 1:**
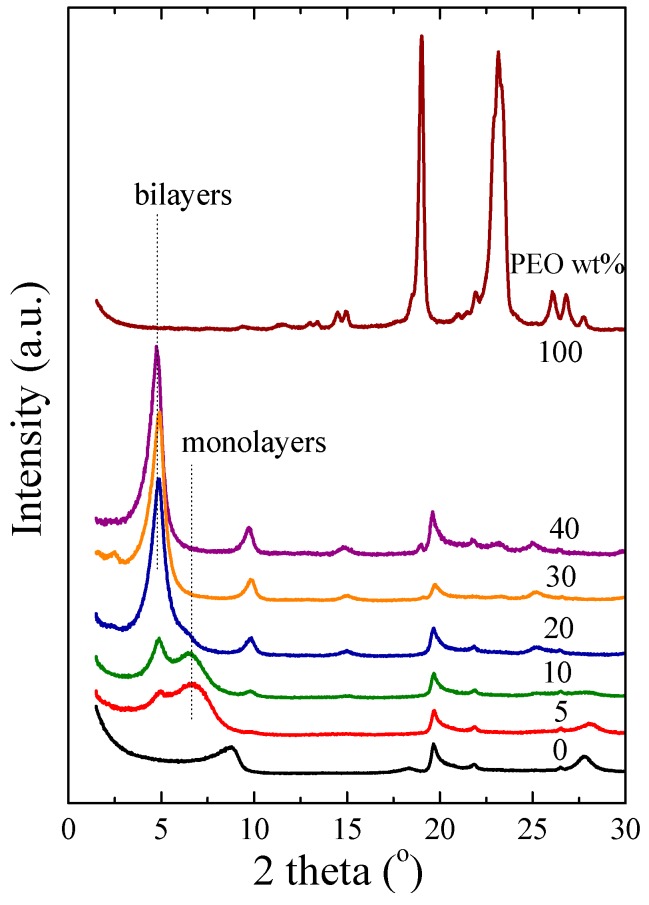
X-ray diffraction measurements of pure PEO, pure Na^+^-MMT, and PEO/Na^+^-MMT nanocomposites with 5–40 wt % PEO. The curves have been shifted for clarity.

Figure 1 shows X-ray diffraction measurements of pure Na^+^-MMT, pure PEO and nanocomposites with polymer content between 5 and 40 wt %. Pristine montmorillonite possesses a main characteristic diffraction peak (*001*) at 2θ = 8.8°, which is attributed to the 1 nm interlayer distance, estimated by Bragg’s law. PEO at room temperature is in its crystalline form and its diffractogram is characterized by a series of diffraction peaks, the most important of which are observed at 2θ = 19.0° and 23.2° corresponding to periodic distances of 0.46 and 0.38 nm, respectively [77,78]. In the majority of the studies in the literature, PEO crystalizes according to the 7_2_ helical model, which means that seven monomeric units turn two times per fiber period. The structure is found appreciably distorted compared to one with exact helical symmetry with the distortion attributed to the flexibility of the molecular chain and the intermolecular forces [79]. On the other hand, a planar zig-zag crystal structure was reported for PEO after drawing about two-fold after necking at room temperature [80]. Additionally, various configurations have been reported in PEO crystalline complexes with urea, thiurea, and HgCl_2_, amplifying the assumption of the flexibility of the PEO molecule [79].

The diffractograms of the two nanohybrids with the higher polymer content (30 and 40 wt %) show one main diffraction peak at low diffraction angles. It is observed at 2θ = 4.8° and it is attributed to the periodic structure of the montmorillonite; the peak has been shifted to lower angles due to the increased interlayer distance of the inorganic material caused by the intercalation of the poly(ethylene oxide) chains. The resulting interlayer distance is 1.85 nm and it corresponds to the formation of bi-layers of polymer chains inside the galleries. The peaks at 2θ = 9.7° and 2θ = 14.8° are the higher order diffraction peaks observed due to the substantial coherence of the intercalated structure. At lower polymer compositions, another peak is observed at 2θ = 6.7° corresponding to *d_001_* = 1.3 nm, which denotes the existence of monolayers in the galleries as well; increase of the polymer content leads to the decrease of the peak that corresponds to the monolayers (at 20 wt % this is seen as a shoulder) and the increase of the peak of the bi-layers. The formation of bi-layers completes approximately at 30 wt % of PEO, which denotes that the intercalated macromolecules have occupied the interlayer volume and that, at compositions higher than ~40 wt %, they remain outside the completely filled galleries covering the outer walls of the inorganic particles. This is in agreement with a simple calculation based on the measured interlayer distances of the pure and intercalated Na^+^-MMT, as well as the densities of PEO (1.13 g/cm^3^ at 25 °C, Sigma Aldrich) and clay (2.86 g/cm^3^, Southern Clay) that results in that the necessary polymer to completely fill the galleries is 20–25 wt %. 

Moreover, for the specific compositions of the nanohybrids studied herein, there is no evidence of any diffraction peaks corresponding to the crystalline structure of PEO, implying that both the intercalated chains and the chains that are in close proximity to the walls of the clay particles are purely amorphous. These results are further verified by differential scanning calorimetry, DSC, measurements [31] that show no thermal transition in the temperature range of PEO melting and/or crystallization for these compositions, verifying the XRD results. Thus, the polymer chains inside the galleries, as well as in close proximity with the outer surfaces of the inorganic surfaces, are amorphous. In a previous work [31] utilizing similar systems over a broader range of compositions, we had focused on the investigation of the crystallinity and on its dependence on hybrid composition utilizing XRD, DSC, Raman and infrared spectroscopy. In that case, it was shown that it was only for polymer concentrations higher than 70 wt %, where a large amount of excess polymer exists outside the intercalated galleries, that polymer crystallinity was observed [31]. 

**Figure 2:**
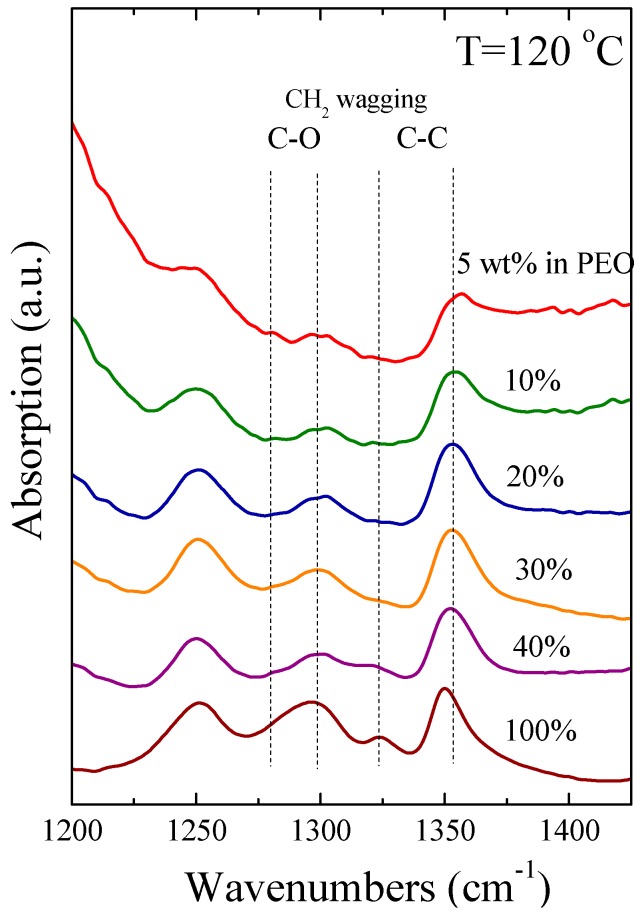
ATR-FTIR Spectra of pure PEO (bottom) and PEO/Na^+^-MMT nanocomposites with varying polymer content. The intensity of the peaks are normalized to the one at 1350 cm^−1^ of the PEO whereas the curves have been shifted for clarity.

Furthermore, a change in the chain conformation of the amorphous polymer was observed for a nanohybrid with 30 wt % PEO [31], with a significant increase of the *gauche* population compared to that in the polymer melt. In the present work, we investigate in detail this change in the conformation as well as its dependence on the nanohybrid composition utilizing the series of nanohybrids, where crystallinity is completely suppressed and the polymer chains are amorphous. Figure 2 shows ATR-FTIR spectra of the nanohybrids together with the respective one of the pure polymer at T = 120 °C, i.e., at a temperature much higher than the melting temperature of PEO; the data are shown in the spectral region where the CH_2_ wagging modes appear. PEO has a repeated unit along the backbone consisting of three linkages O–C, C–C, and C–O. Although *ttt* (*trans*-*trans*-*tran*s) conformation seems to be the more stable considering steric effects alone [81], the most stable configuration is indeed *tgt* (*trans*-*gauche*-*trans*) including a *gauche* conformation for the majority of the C–C bonds [82,83]. The stability of the *tgt* configuration is attributed to its polar character, as the hydrogen atoms point outwards from the center of the helix and the oxygen atoms point inwards. The rotation of C–C and C–O bonds, which modifies the polymer configuration, depends on the interactions with the environment, while characteristics, like polarity and temperature, play a crucial role. Thus, the interactions of the polymer chains with the clay surfaces in a nanocomposite should have a significant impact on the conformations of polymer chains.

In the range of the FTIR spectra shown in Figure 2, the absorption bands centered at 1350 and 1325 cm^−1^ are assigned to the *gauche* and *trans* conformations, respectively, of the C–C bond for the neat PEO, while the peaks located at 1300 and 1285 cm^−1^ are attributed to the *gauche* and *trans* conformation, respectively, of the C–O bond [83,84,85,86,87]. The spectra are normalized using the peak at 1350 cm^−1^, in an attempt to visualize the effect of polymer composition on the ratio of *gauche* to *trans* conformations, as described by the area peak ratio of the 1350 to the 1325 cm^−1^ bands. As can be clearly observed, differences in the polymer conformation can be monitored as the polymer composition decreases. Despite the fact that the peak positions are essentially the same, their relative intensities are significantly modified; it is clear that the intensity of the peak at 1325 cm^−1^ decreases as the concentration of Na^+^-MMT increases, reflecting a corresponding decrease of *trans* configurations and the adoption of *gauche* conformations for the intercalated chains. For the hybrids with the lower polymer compositions, i.e., 5 and 10 wt %, the bands at 1285 and 1325 cm^−1^ assigned to *trans* conformations of the C–O and the C–C bonds are almost completely vanished.

**Figure 3:**
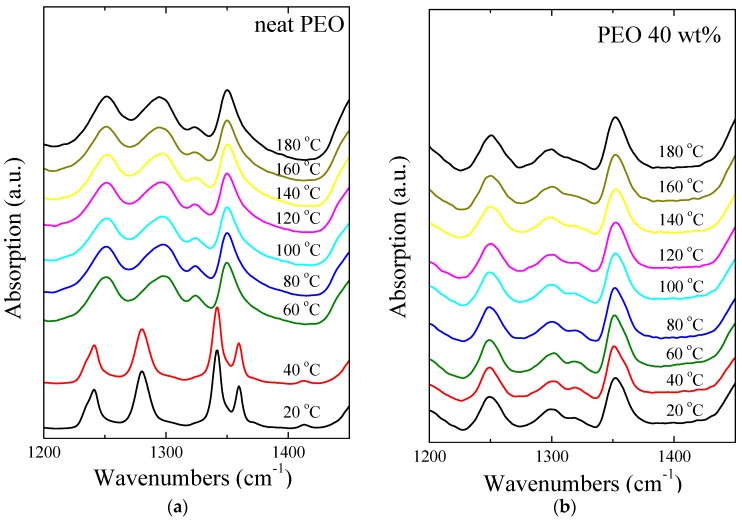
ATR-FTIR spectra of (**a**) neat PEO and (**b**) nanocomposite of PEO/Na^+^-MMT with 40 wt % in PEO at different temperatures. The curves have been shifted for clarity.

ATR-FTIR measurements were performed for all specimens over a broad temperature range covering the regime from room temperature to much higher than the pure polymer melting temperature. Figure 3 shows such measurements for the bulk PEO (Figure 3a) and for a nanohybrid with 40 wt % (Figure 3b) polymer. All samples were heated to 180 °C, with a temperature step of 20 °C, in inert atmosphere and the relative spectra were collected after leaving the material at each temperature for 5 min to ensure that the specimen temperature is uniform. For the neat PEO at 20 and 40 °C, the presence of typical bands at 1340 and 1360 cm^−1^ evidence the presence of crystalline phase [88]. It should be noted that such bands due to the crystalline phase are completely absent in the spectra of the nanocomposite for all temperatures investigated. As depicted in Figure 3a, the crystalline bands of neat PEO are replaced by an amorphous band at 1350 cm^−1^ for temperatures higher than 60 °C. In this context, the presence of a broad peak roughly centered at 1350 cm^−1^, which corresponds to the amorphous phase for all spectra in Figure 3b, indicates that the polymer is amorphous even at temperatures much below the melting point of bulk PEO. The findings are very similar for all the bands of the various vibrations and not only the one attributed to the CH_2_ wagging shown in Figure 3; the sharp peaks that are observed in the PEO spectrum at temperatures below the melting temperature are absent from the nanohybrid spectra for all temperatures (and of course from the neat PEO spectra at high temperatures). Exactly similar behavior is observed for all of the nanohybrids of the present investigation.

**Figure 4:**
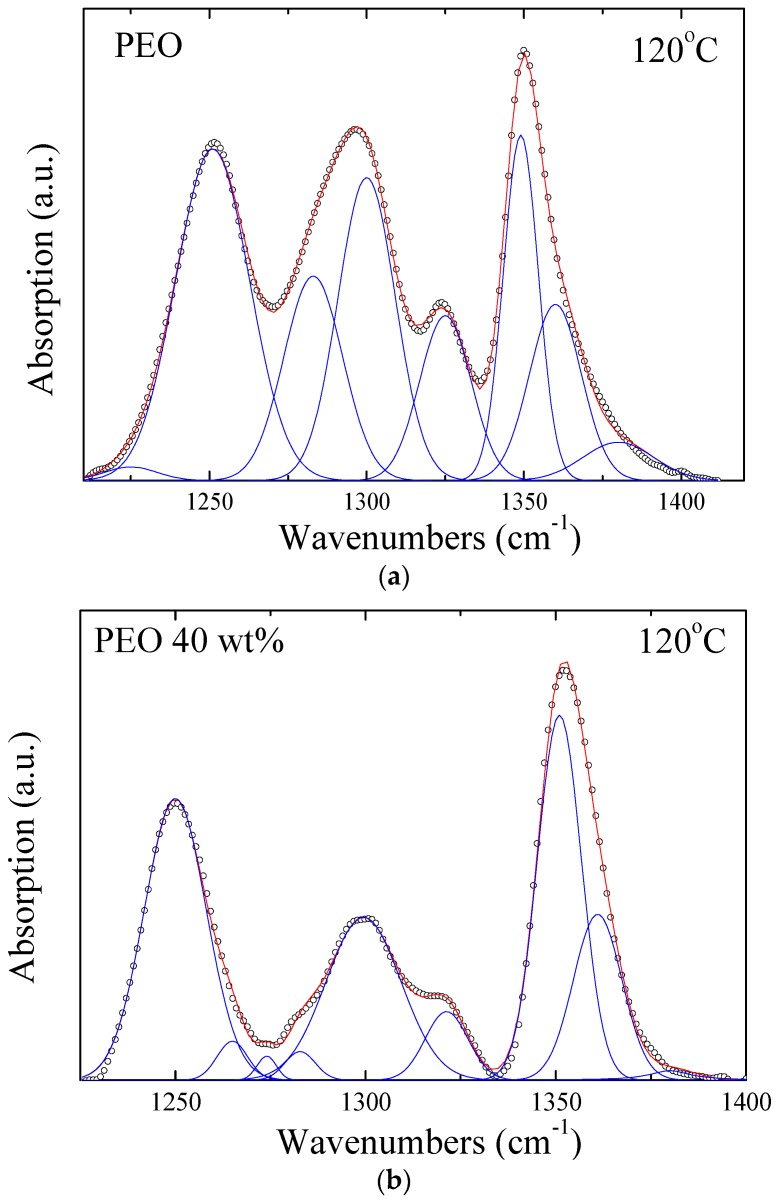
Fitting of ATR-FTIR Spectra of (**a**) pure PEO and (**b**) PEO/Na^+^-MMT with 40 wt % in PEO at 120 °C. The lines denote the individual Gaussian peaks as well as the total fit.

A thorough analysis of the obtained FTIR spectra took place in order to extract the information about the chain conformations of the polymer in the bulk and in the nanocomposites. More specifically, careful deconvolution of the peaks in the region of 1225 to 1400 cm^−1^ was performed utilizing a series of Gaussian peaks. As mentioned earlier for the neat PEO, the absorption bands that correspond to the C–C bond are centered at 1350 and 1325 cm^−1^ for the *gauche* and *trans* conformation, while the absorption bands that correspond to the C–O bond are located at 1300 and 1285 cm^−1^ for the *gauche* and *trans* conformations, respectively. The bands for the nanocomposite are found at approximately the same positions. A representative example of the obtained results is presented in Figure 4a for neat PEO at 120 °C, while Figure 4b shows the corresponding result of the nanocomposite with 40 wt % PEO at the same temperature for comparison. It is noted that two additional bands centered at 1265 and 1275 cm^−1^ of low intensities were necessary to be included in order to successfully deconvolute the spectrum of the nanohybrid. Moreover, small peaks on the left and on the right side of the selected region of the spectra were used for refining the fitting curve. By comparing the spectra of the two materials of Figure 4, it becomes obvious that the peak corresponding to the *trans* conformation in the spectra of the nanohybrid is significantly smaller than the respective one in the spectra of the neat polymer and, thus, the ratio of *gauche* to *trans* conformations for the C–C bond clearly increases. The same conclusion can be derived by studying the configuration of the C–O bond. The *trans* conformations of the C–O bond decrease compared to the respective ones in neat PEO. At the same time, the corresponding shoulder tends to separate from the main absorption band at 1300 cm^−1^, which is related to the *gauche* configuration of the C–O bond. The influence of the presence of the surfaces as well as of the severe confinement they impose to the polymer chains have a clear effect on the configurations of the chains with the increase of the *gauche* population in the hybrid systems being a significant result.

**Figure 5:**
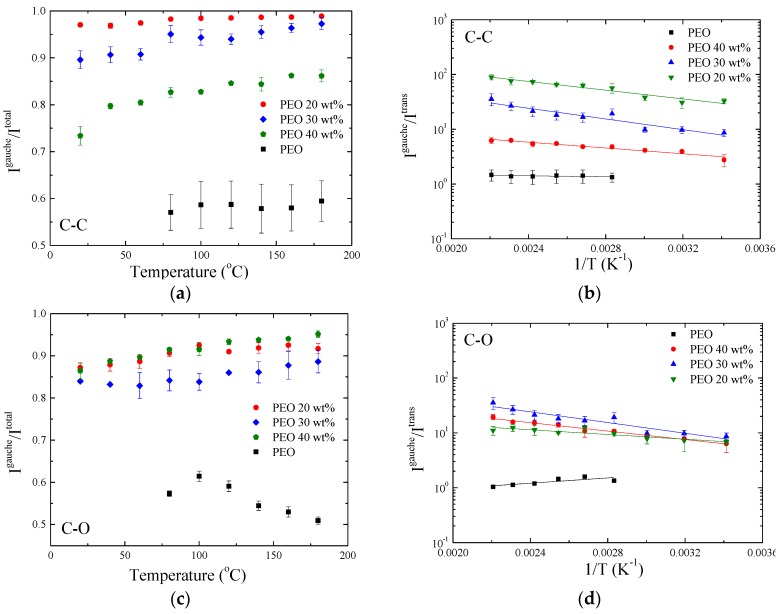
Temperature dependence of the intensity ratio (**a**) *I*^1350^/(*I*^1325^ + *I*^1350^) and (**c**) *I*^1300^/(*I*^1285^ + *I*^1300^) signifying the ratio *I^gauche^/I^total^* for pure PEO and nanocomposites of varying compositions. In the right part the ratios (**b**) *I*^1350^/*I*^1325^ and (**d**) *I*^1300^*I*^1285^ signify the ratio *I^gauche^/I^trans^* in an Arrhenius representation for the respective samples.

The tendency of PEO to adopt mainly *gauche* conformation in the nanocomposites seems to become more pronounced with increasing temperature. On the contrary, temperature slightly affects the conformation of the neat polymer. The effect of temperature on the chain configuration was investigated more quantitative by studying the ratio *I^gauche^/I^total^* and *I^gauche^/I^trans^* for both the C–C and the C–O bonds. Figure 5a shows the ratio of the integrated intensities *I*^1350^/(*I*^1325^ + *I*^1350^) for the C–C bond of the pure PEO and the nanocomposites with 20, 30, and 40 wt % PEO content, whereas Figure 5c shows the ratio *I*^1300^/(*I*^1285^ + *I*^1300^) for the C–O bond for the respective samples. It is noted that, for nanohybrids with lower polymer content, the analysis and the calculation of such ratios was not possible since the peaks corresponding to the *trans* conformations were completely suppressed. In both cases, it is clear that for the neat polymer almost half of the conformation are in the *gauche* state with the other half being *trans*. A significant change is observed for the nanohybrids, where the increase of the *gauche* conformation is clearly evident. More specifically, for the C–C bond it seems that this increase is systematic with composition and that the *gauche* conformation increases as the polymer content decreases. For the C–O bond, however, the increase of the *gauche* population does not show a clear composition dependence. Moreover, the fraction of *gauche* population for the C–C bond of the neat PEO is basically constant with increasing temperature whereas the respective ratio for the C–O bond tends to decrease with temperature, probably because of steric interactions between adjoining methylene groups [89,90]. In the case of the nanocomposites, the fraction of *gauche* conformation for both C–C and C–O bonds increases with temperature.

Figure 5b,d show the ratios *I*^1350^/*I*^1325^ and *I*^1300^/*I*^1285^, signifying the ratio *I^gauche^/I^trans^* for the C–C and the C–O bonds, respectively, in an Arrhenius representation. The preference of the polymer chains in the nanocomposites to adopt *gauche* conformations is clear in this representation, as well. In order to quantify the results of the analysis, the energy difference between the state of *gauche* and *trans* conformations was determined utilizing the Arrhenius representation. Fitting of the data of Figure 5b results in that, for the C–C bond of PEO, the energy of the *gauche* state is 0.19–0.32 kcal/mol lower than the respective of the *trans* state. The small energy difference is in agreement with the observation that the polymer conformations are divided almost equally between the *gauche* and *trans* states. In the case of the nanohybrids, this energy difference becomes ΔG = G_t_ − G_g_ = 0.66 to 1.69 kcal/mol for the 40 wt %, ΔG = 1.16 to 3.01 kcal/mol for the 30 wt % and ΔG = 1.97 to 4.04 kcal/mol for the 20 wt % PEO hybrids. It is noted that, the temperature range of the fitting in the case of the nanocomposites is larger than in the case of the pure polymer since PEO in the hybrids is amorphous even at temperatures below the crystallization temperature of bulk PEO. The systematic increase of the fraction of *gauche* conformations with increasing the amount of clay is evident. The situation is a bit different when the C–O bond is concerned. In this case, the energy difference is Δ*G* = *G*_t_ − *G*_g_ = 1.1–2.6 kcal/mol for the 40 wt %, Δ*G* = 1.2 to 3.1 kcal/mol for the 30 wt % and Δ*G* = 1.1 to 2.3 kcal/mol for the 20 wt % PEO hybrids. Thus, in contrast to the case of the C–C bond, for the C–O bond there is not any significant dependence of the activation energy on the nanocomposite composition. 

## 4. Conclusions

X-ray diffraction and attenuated total reflectance-Fourier transform infrared spectroscopy have been utilized for the investigation of the structure and conformation of poly(ethylene oxide) chains in intercalated structures of polymer/layered silicate nanocomposites. In all cases where the polymer chains are either confined within the galleries or in close proximity to the outer surfaces of the inorganic particles, the polymer is purely amorphous. A significant change in the conformation of the confined or adsorbed polymer chains in comparison to the ones of the pure PEO is observed. This is evident by the dramatic increase of the *gauche* conformations relative to the *trans* ones for both the C–C and the C–O bonds. There is a difference, however, between the two cases: for the C–C bond, the fraction of the *gauche* population is much higher than that of the pure polymer and it increases with decreasing polymer content whereas, for the C–O bond, the fraction of *gauche* conformation is much higher than that of the pure polymer but it does not change with composition of the nanohybrids.

## Abbreviations

The following abbreviations are used in this manuscript:

PEO	poly(ethylene oxide)
Na^+^-MMT	Sodium Montmorillonite
ATR-FTIR	Attenuated Total Reflectance-Fourier Transform Infrared Spectroscopy
XRD	X-ray Diffraction
DSC	Differential Scanning Calorimetry
CEC	Cation Exchange Capacity
*ttt*	*trans*-*trans*-*trans*
*tgt*	*trans*-*gauche*-*trans*
